# Use of selected samples to diagnose a tricky feline viral disease in a cat with uveitis and neurological signs

**DOI:** 10.29374/2527-2179.bjvm001223

**Published:** 2023-07-24

**Authors:** Julio Cesar Neves de Almeida, Heloísa Cristina Teixeira de Carvalho, Lana Isabella Gila, Nathana Beatriz Martins, Matias Pablo Juan Szabó, Aline Santana da Hora

**Affiliations:** 1 Veterinarian, Programa de Pós-Graduação em Ciências Veterinárias (PPGCV), Universidade Federal de Uberlândia (UFU). Umuarama, Umuarama, MG, Brazil; 2 Veterinarian, MSc., Programa de Pós-Graduação em Ciências Veterinárias (PPGCV), Universidade Federal de Uberlândia (UFU). Umuarama, Umuarama, MG, Brazil; 3 Veterinarian, DSc., Laboratório de Patologia Animal, UFU. Umuarama, Umuarama, MG, Brazil; 4 Veterinarian, DSc. Laboratório de Investigação Etiológica Veterinária UFU. Umuarama, Umuarama, MG, Brazil

**Keywords:** molecular diagnosis, feline immunodeficiency virus, feline leukemia virus, feline infectious peritonitis virus, diagnóstico molecular, vírus da imunodeficiência felina, vírus da leucemia felina, vírus da peritonite infecciosa felina

## Abstract

This case involved a 2-year-old neutered male domestic mixed-breed cat that was rescued from the street eight months earlier. The animal presented with weakness, hyporexia, progressive weight loss, fatigue, uveitis, pale mucous membranes, dehydration (7%), and pelvic limb paresis. Aqueous humor was collected for molecular analysis for the differential diagnosis of potential etiological agents [Feline coronavirus (FCoV), Feline leukemia virus (FeLV), Feline immunodeficiency virus (FIV), *Toxoplasma gondii*, *Cryptococcus* spp., *Felid herpesvirus*-1 (FHV-1) and *Bartonella* spp.] of feline uveitis. The sample was positive by real-time reverse transcription-polymerase chain reaction (RT-qPCR) for FCoV and RT-qPCR and real-time polymerase chain reaction (qPCR) for FeLV and qPCR FIV. The cat was euthanized due to poor clinical outcomes and prognosis. A cerebrospinal fluid (CSF) sample was collected and tested, and the same pathogens were found in the aqueous humor. Small-cell follicular multicenter lymphoma and multifocal pyogranulomatous meningoencephalitis were observed upon histopathological analysis. In this study, aqueous humor and cerebrospinal fluid samples were efficient for the detection of coinfection with FIV, FeLV, and FCoV.

## Resumo

O caso refere-se a um gato de dois anos de idade, sem raça definida, resgatado da rua há oito meses. O animal apresentava fraqueza, hiporexia, emagrecimento progressivo, cansaço fácil, uveíte, mucosas pálidas, desidratação (7%) e paresia de membros pélvicos. O humor aquoso foi coletado para o diagnóstico molecular diferencial de potenciais agentes etiológicos [coronavírus felino (FCoV), vírus da leucemia felina (FeLV), vírus da imunodeficiência felina (FIV), *Toxoplasma gondii*, *Cryptococcus* spp., herpesvírus felino tipo 1 (FHV-1) and *Bartonella* spp.] causadores de uveíte felina. A amostra foi positiva na reação em cadeia da polimerase precedida por transcrição reversa em tempo real (RT-qPCR) para FCoV, RT-qPCR e reação em cadeia da polimerase em tempo real (qPCR) para FeLV e qPCR para FIV. O animal foi submetido à eutanásia - devido ao quadro clínico e prognóstico desfavorável. Amostra de líquido cefalorraquidiano (LCR) foi coletada e testada, confirmando a identificação dos mesmos patógenos encontrados no humor aquoso. Linfoma multicêntrico folicular de pequenas células e meningoencefalite piogranulomatosa multifocal foram observados na análise histopatológica. Neste relato, as amostras de humor aquoso e líquido cefalorraquidiano foram eficientes para a detecção de coinfecção por FIV, FeLV e FCoV.

## Introduction

In cats, infectious agent-induced endogenous uveitis is the most frequent cause of uveitis, and the most frequently recorded infectious agents are the Feline coronavirus (FCoV), Feline immunodeficiency virus (FIV), and Feline leukemia virus (FeLV) (Jinks et al., 2016). However, other pathogens, such as *Toxoplasma gondii*, *Cryptococcus* spp., Felid herpesvirus-1 (FHV-1), and *Bartonella* spp. (Powell et al., 2010), may be associated with uveitis. Determining the specific pathogen causing uveitis can be challenging owing to similar clinical signs and difficulty in diagnosing certain pathogens.

Pathogens are often located at the sites where they are causing injury, without circulation in the bloodstream, which restricts the diagnosis to the use of samples selected according to clinical manifestations. A great example of this is FCoV, which causes the dry form of feline infectious peritonitis (FIP) (Tasker, 2018).

To accurately diagnose the cause of uveitis in cats, it is necessary to collect aqueous humor, which, despite being well established and described in specialized literature (Featherstone et al., 2021), is not practiced by clinicians who are often unfamiliar with the technique or who are simply afraid to perform it. In addition, there are few reports on the use of aqueous humor as a fundamental sample for diagnosing infectious diseases in cats.

This case report aimed to describe the ante-mortem molecular diagnosis of feline uveitis in a cat, highlighting the importance of aqueous humor as an elective sample for the accurate and differential diagnosis of uveitis.

## Case report

A 2-year-old neutered male domestic mixed-breed cat, weighing 1.8 kg, was admitted to a veterinary hospital after being rescued eight months prior. During the rescue, the cat tested positive for FeLV and negative for FIV using an immunochromatographic test (FIV Ac/FeLV Ag Test Kit, Alere). At presentation, the animal showed weakness and hyporexia for two days, progressive weight loss and fatigue for one week, and generalized hair loss for two months. Four asymptomatic cats that tested positive for FeLV lived with the animal. All cats had access to the street and were not vaccinated.

On physical examination, the animal presented with pale and mildly jaundiced oral and ocular mucosa, 7% dehydration, a body score of 3/9, paresis of the pelvic limbs, and uveitis.

On complete blood count, the patient presented anemia (hematocrit 14.2%; reference interval [RI] 24-45%), a normal total leukocyte count (14,300 mm^3^; RI 5,500-19,500 mm^3^), an increased number of band neutrophils (715 mm^3^; RI 0-300 mm^3^), and normal lymphocyte count and thrombocytopenia (200 × 10^3^/µL; RI 230.000-680.000 × 10^3^/µL). Macroplatelets, reactive lymphocytes, and monocytes were observed during the cytological evaluation. Hypoalbuminemia (1.84 g/dL; RI 2.1-3.3 g/dL), hyperproteinemia (11.0 g/dL; RI 6.0-8.0 g/dL), and a low albumin: globulin ratio (A:G) of 0.16 were found. The serum biochemical tests performed showed normal levels of alkaline phosphatase (25 U/L; RI 26-93 U/L), alanine aminotransferase (46 U/L; RI 6.0-83 U/L), and creatine (0.83 mg/dL; RI 0.8-1.8 mg/dL). However, the gamma glutamyl transferase level was high (10 U/L; RI 1.5-5.3 U/L) and macroscopically, the serum appeared icteric.

The ophthalmic examination revealed uveitis in the left eye, with fibrin deposition in the anterior chamber and a brownish color in the iris compared to the normal contralateral eye. Therefore, aqueous humor was collected to perform a molecular diagnostic panel for the main infectious causes of uveitis in cats.

The cat was anesthetized with midazolam [0.02 mg/kg, IM (DORmire, Cristália)] and methadone [0.1 mg/kg, IM (MYTedom, Cristália)], and maintained with isoflurane (Isoflorine, Cristália). Eye antisepsis was achieved using 5% iodopovidone (Septmax) followed by a sterile saline solution in the conjunctival sac and bulbar conjunctiva. Aqueous humor was collected using the paracentesis technique of the anterior chamber according to Featherstone et al. (2021). No adverse events were observed in the cat after this collection.

Total RNA and DNA were extracted from the aqueous humor sample (0.2 mL) using the PureLink Viral RNA/DNA Mini Kit (Invitrogen, Thermo Fisher Scientific) according to the manufacturer’s protocol. The sample was analyzed for RNA (FCoV and FeLV), DNA pathogens (*T. gondii*, *Cryptococcus* spp., FHV-1, and *Bartonella* spp.), and pro-viral DNA (FeLV and FIV). For FeLV RNA detection, 8 μL of the total extracted RNA was treated with 1U DNase (RQ1 RNase-Free DNase, Promega), and subsequently submitted to phenol/chloroform and ethanol precipitation, according to the manufacturer’s instructions. All primers used were obtained from previously published studies (Table 1). For all reactions, GoTaq® qPCR and 1-Step RT-qPCR Master Mixes (Promega) were used, according to the manufacturer’s instructions, in a Rotor-Gene (Qiagen) real-time thermocycler. Ultrapure nuclease-free water (Promega) was used as a negative control, and all positive controls are presented in Table 1.

**Table 1:** Pathogens, region or gene target, and reference and positive control used in molecular assays.

**Pathogen**	**Region/gene target**	**Reference**	**Positive control**
***Bartonella* spp.**	ssrA gene	Duplan et al., 2018	Spleen sample from *Euphractus sexcinctus*
***Cryptococcus* spp.**	ITS rDNA region	Gago et al., 2014	Nasal cytology sample (imprint) from a cat with cryptococcosis
**FCoV**	7b gene	Gut et al., 1999	Abdominal effusion sample from a cat with FIP
**FeLV**	U3 region of the long terminal repeat (LTR)	Tandon et al., 2005	Blood sample from a cat with progressive FeLV
**FHV-1**	Thymine kinase (TK) gene	Burgesser et al., 1999	Feline-4 vaccine (Boehringer-Ingelheim)
**FIV**	*gag* gene	Wang et al., 2010	Blood sample from a FIV-positive cat
***T. gondii***	18S rDNA region	Lalonde and Gajadhar, 2011	RH-B strain

Note. FCoV, *Feline coronavirus*; FeLV, *Feline leukemia virus*; FHV-1, *Felid herspevirus*-1; FIV, *Feline immunodeficiency virus*; *T. gondii*, *Toxoplasma gondii*.

The presence of RNA from FCoV and FeLV and pro-viral DNA from FeLV and FIV was detected in the aqueous humor, and the animal was euthanized because of the unfavorable clinical picture and prognosis. The euthanasia was performed with propofol (Propovan, Cristália, 30 mg/kg, IV) before potassium chloride (KCl 19,1%, Isofarma, 75 mg/kg, IV) administration. A sample of cerebrospinal fluid (CSF) was collected, qPCR and RT-qPCR were performed for the same pathogens as described previously, and identical coinfection was identified in this sample as in the aqueous humor. Based on these results, the FeLV infection was classified as progressive.

**Figure 1:**
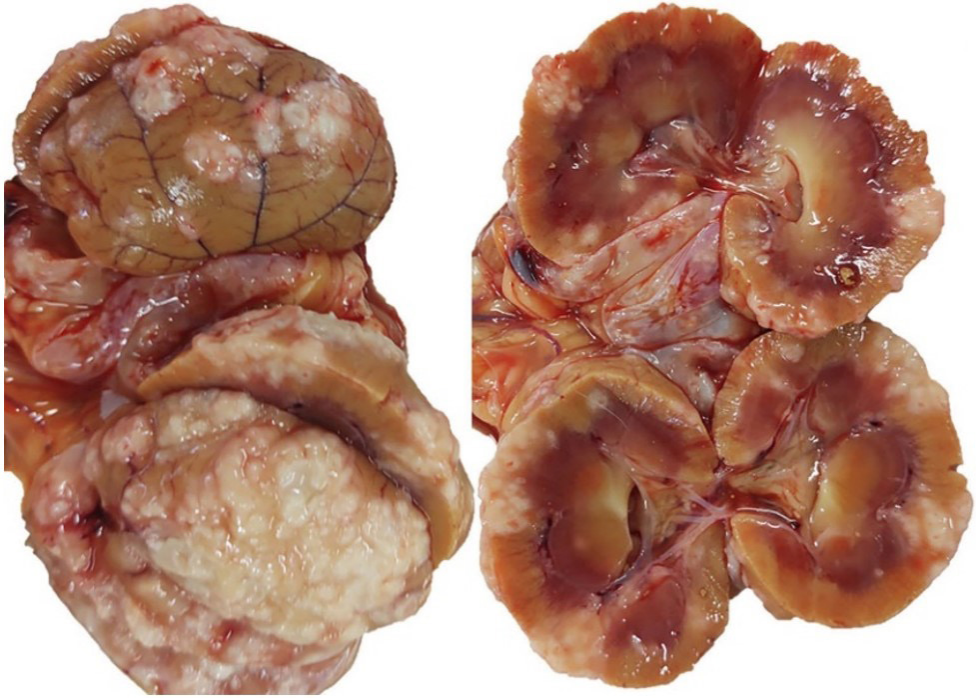
Two-year-old neutered male domestic mixed breed cat kidney alterations under macroscopic examination. Note. The cat was coinfected with Feline coronavirus (FCoV), Feline immunodeficiency virus (FIV), and Feline leukemia virus (FeLV). The kidneys have irregular surfaces owing to the presence of multiple prominent and whitish millimetric nodules. The nodules are predominantly located in the renal cortex.

During necroscopic examination, samples from the prescapular lymph nodes, spleen, kidneys, brain, cerebellum, and left eyeball were collected and subjected to histopathological examination. The collected organs were fixed in 10% buffered formaldehyde, trimmed, embedded in paraffin, sectioned at 5 μm, stained with hematoxylin and eosin, and analyzed using optical microscopy. The prescapular lymph nodes were slightly increased in volume and pale. The spleen moderately increased in volume with an irregular surface, multiple firms, and whitish millimetric nodules that entered the splenic parenchyma. The kidneys had an irregular surface due to multiple prominent, firm, whitish, and multifocal-to-coalescing millimetric nodules located predominantly in the region of the renal cortex (Figure 1). The brain and cerebellum did not present any evident macroscopic alterations, with only flattened gyri, shallow sulci, and engorged meningeal vessels.

**Figure 2:**
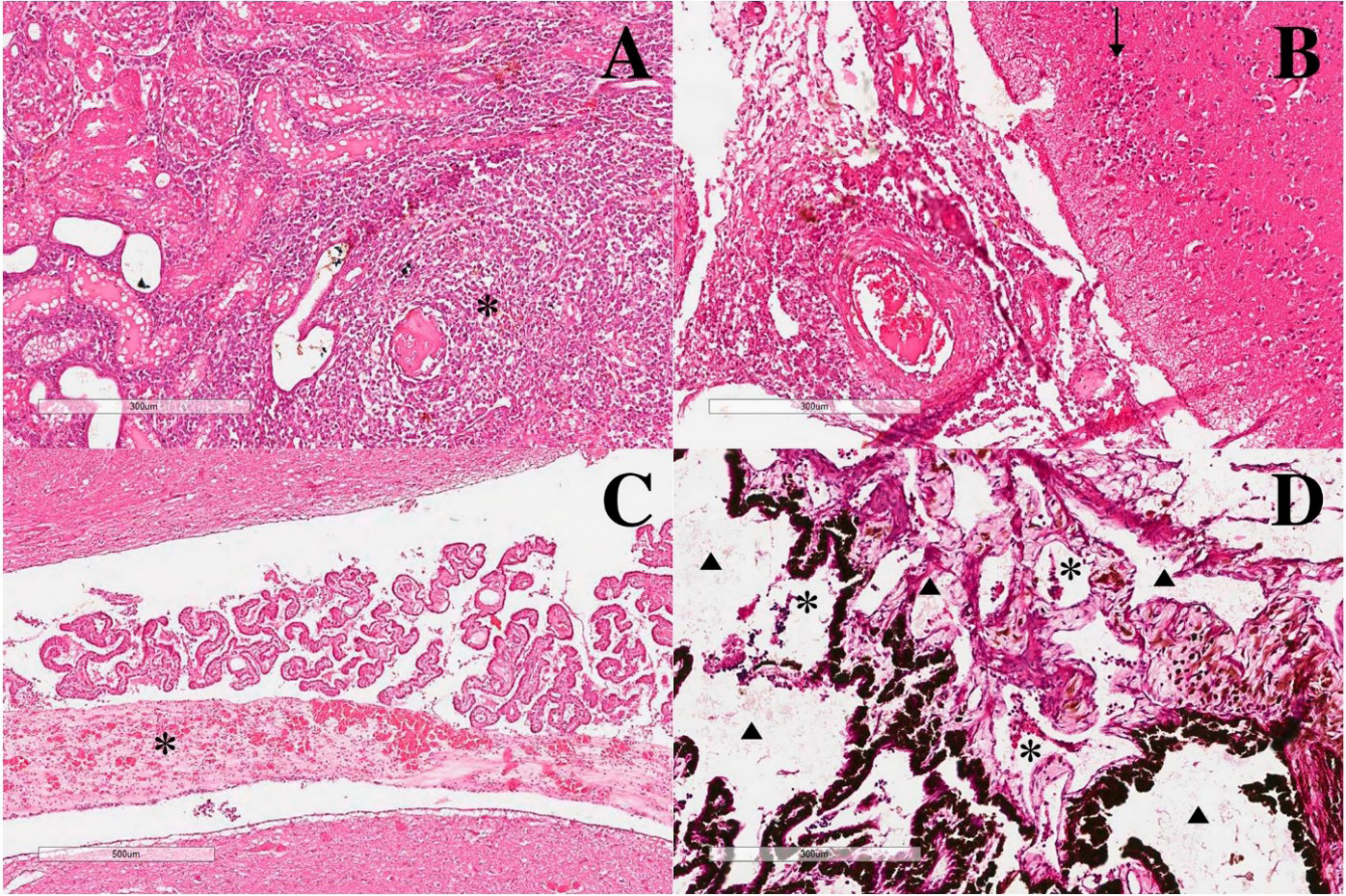
Kidney, brain, and eye histopathology of a 2-year-old neutered male domestic mixed breed cat. Note. The cat was coinfected with Feline coronavirus (FCoV), Feline immunodeficiency virus (FIV), and Feline leukemia virus (FeLV). (A) Kidney histopathology. Loss of architecture due to the monomorphic neoplastic proliferation of small lymphocytes (asterisk). Hematoxylin and eosin (H&E) staining. (Magnification 100×, bar = 300 µm). (B) Brain histopathology. The meningeal artery showed dissociation of smooth muscle fibers and moderate adjacent pyogranulomatous inflammatory infiltrates. In the brain parenchyma, a cluster of glial cells was observed, forming a discrete glial nodule (arrow). H&E stain. (Magnification 100×, bar = 300 µm). (C) Fourth ventricle histopathology. The fourth ventricle contains red blood cells and an inflammatory infiltrate of lymphocytes, macrophages, and neutrophils associated with the fibrin mesh (asterisk) adjacent to ependymal cells. H&E stain. (Magnification 50×, bar = 500 µm). (D) Histopathological examination of the eyes. The ciliary body with increased space between smooth muscle fibers associated with discrete lymphocytic inflammatory infiltrates, red blood cells (asterisk), and fibrin (triangle). H&E stain. (Magnification 100×, bar = 300 µm).

The histopathology of lymph nodes, spleen, and kidney samples revealed monomorphic neoplastic proliferation of small lymphocytes in a follicular pattern. The diagnosis was small-cell follicular multicentric lymphoma (Figure 2A) according to Morton et al. (2014). The brain sample presented pia mater with moderate focal pyogranulomatous vasculitis, characterized by the dissociation of smooth muscle fiber arteries, alteration of the tunica intima, and moderate infiltration of lymphocytes, macrophages, and neutrophils (Figure 2B). Discrete lymphocytic perivascular cuffing was observed, suggesting discrete multifocal lymphocytic encephalitis. Discrete clusters of glial cells with a multifocal distribution in the brain parenchyma (glial nodules) were also observed. The pia mater in the cerebellum exhibited moderate inflammatory infiltration of lymphocytes, rare macrophages, and neutrophils, suggesting moderate multifocal pyogranulomatous meningitis. Moderate numbers of red blood cells, lymphocytes, macrophages, and neutrophils associated with fibrin and adjacent to ependymal cells were observed in the fourth ventricle, suggesting moderate pyogranulomatous ventriculitis (Figure 2C). In the ocular globe, the dissociation of smooth muscle fibers from the ciliary body region and the increased space between them indicated edema. Discrete lymphocytic inflammatory infiltrates, rare red blood cells, and moderate levels of fibrin suggested discrete uveitis (Figure 2D).

## Discussion

This study reports an unprecedented diagnosis of non-effusive FIP with coinfection of FIV and FeLV by RT-qPCR and qPCR in aqueous humor (ante-mortem) and CSF (post-mortem) samples. The choice of appropriate samples for molecular diagnosis is extremely important for diagnostic sensitivity. Therefore, the preferred samples for the present study were selected based on the presence of uveitis and neurological clinical signs.

The detection of FCoV RNA, using RT-PCR, in tissues, effusions, CSF, and aqueous humor is strongly indicative of FIP (Tasker, 2018). The presence of FCoV genetic material in the aqueous humor of cats with FIP has been reported (Sangl et al., 2020).

In a study, the specificity obtained by RT-qPCR with aqueous humor was 100%, while the sensitivity was 35.5% in samples obtained postmortem, which possibly resulted in some degradation of viral RNA (Sangl et al., 2020). However, in this report, the paracentesis of the aqueous humor was performed *in vivo* and the sample was immediately sent to the laboratory in a thermal box on ice to avoid the degradation of the material.

Aqueous humor samples have also been used to diagnose infectious uveitis by PCR for the following etiological agents: *Bartonella* spp., Felid herpesvirus type 1 (FHV-1), *T. gondii* (Powell et al., 2010), and FIV (Ryan et al., 2003). Ryan et al. (2003) experimentally infected 12 cats intravenously and identified the presence of FIV genetic material in three aqueous humor samples and eight CSF samples in the acute phase of infection, which was characterized by a transient increase in viral plasma loads, followed by a decrease in loads with minimal virus detection. These findings are consistent with those of the present study, in which both samples were positive for pro-viral FIV DNA.

The animal in this report presented nonspecific clinical signs compatible with FIP (Tasker, 2018), FeLV, and FIV (Little et al., 2020) infections, as well as lymphoma (Beatty, 2014). Increased kidney volume on palpation and paresis of the pelvic limbs are often associated with non-effusive FIP, which may present with neurological signs and/or ophthalmopathy, such as uveitis, and is associated with granulomatous lesions that affect various tissues, including the kidneys (Tasker, 2018). Some findings, such as uveitis and pelvic limb paresis, may also be present in cases of progressive infection caused by FeLV (Hartmann & Hofmann-Lehmann, 2020; Little et al., 2020) and in animals with FIV in the terminal phase (Little et al., 2020). However, it is not possible to rule out the possibility that this cat was in the acute phase of FIV infection.

The hematological and biochemical abnormalities observed in this case could indicate various feline diseases, including viral infections such as FeLV, FIV, and FCoV, as well as multicentric lymphoma. Although cats with FIV and FeLV commonly have a low A:G ratio (Jeffery et al., 2012), a ratio below 0.4 strongly suggests FIP (Felten & Hartmann, 2019). Thus, an A:G ratio of 0.16 is the most prominent biochemical laboratory abnormality observed in the cat examined in the present study, thereby indicating a specific viral infection, specifically FIP.

The histopathological analysis of this cat showed nodal and renal lymphoma, with renal and hyaline tubular degeneration, compatible with progressive FeLV infection (Hartmann & Hofmann-Lehmann, 2020). However, due to their oncogenic potential, extranodal lymphomas can also be found in animals with FIV (Little et al., 2020).

Moderate multifocal pyogranulomatous meningitis, moderate pyogranulomatous ventriculitis in the cerebellum, discrete multifocal lymphocytic encephalitis, pyogranulomatous vasculitis, edema, and cerebral congestion were also observed. Pyogranulomatous inflammation of the central nervous system has often been described in cases of non-effusive FIP (Crawford et al., 2017; Foley et al., 1998; Mesquita et al., 2016). Mesquita et al. (2016) observed a glial response in the central nervous system of cats with FIP, but no multifocal glial nodules were observed. Additionally, Foley et al. (1998) evaluated FIP with neurological signs in 16 cats, five of which presented predominantly ocular manifestations, such as anterior uveitis.

There have been case reports of FIP and FIV coinfection, diagnosed by renal immunohistology (Cannon et al., 2005). Additionally, there have been cases of FeLV, in which the presence of FCoV was also found in blood samples tested by RT-qPCR (Powers et al., 2018). It is important to note that blood samples are not considered sensitive for the diagnosis of FIP (Pedersen, 2014). However, the present report is the only one in which there was coinfection of both retroviruses with non-effusive FIP in aqueous humor and cerebrospinal fluid samples.

## Conclusion

The precise diagnosis of the etiological agent in serious diseases helps determine the best course of action; to this end, the proper choice of samples is a crucial factor. In this study, aqueous humor and cerebrospinal fluid samples were efficient in detecting coinfection with FIV, FeLV, and FIP. In addition, the collection of both types of samples can be performed *in vivo* and acts as a determinant in difficult diseases such as non-effusive FIP.
